# The function of miR-143, miR-145 and the MiR-143 host gene in cardiovascular development and disease

**DOI:** 10.1016/j.vph.2018.11.006

**Published:** 2019-01

**Authors:** Francesca Vacante, Laura Denby, Judith C. Sluimer, Andrew H. Baker

**Affiliations:** a Centre for Cardiovascular Science, The Queen's Medical Research Institute, Edinburgh EH16 4TJ, UK; b Maastricht University Medical Centre, Dept. of Pathology, Maastricht 6229 HX, The Netherlands

## Abstract

Noncoding RNAs (long noncoding RNAs and small RNAs) are emerging as critical modulators of phenotypic changes associated with physiological and pathological contexts in a variety of cardiovascular diseases (CVDs). Although it has been well established that hereditable genetic alterations and exposure to risk factors are crucial in the development of CVDs, other critical regulators of cell function impact on disease processes. Here we discuss noncoding RNAs have only recently been identified as key players involved in the progression of disease. In particular, we discuss micro RNA (miR)-143/145 since they represent one of the most characterised microRNA clusters regulating smooth muscle cell (SMC) differentiation and phenotypic switch in response to vascular injury and remodelling. MiR143HG is a well conserved long noncoding RNA (lncRNA), which is the host gene for miR-143/145 and recently implicated in cardiac specification during heart development. Although the lncRNA-miRNA interactions have not been completely characterised, their crosstalk is now beginning to emerge and likely requires further research focus. In this review we give an overview of the biology of the genomic axis that is miR-143/145 and MiR143HG, focusing on their important functional role(s) in the cardiovascular system.

## Graphical abstract

**Figure:**
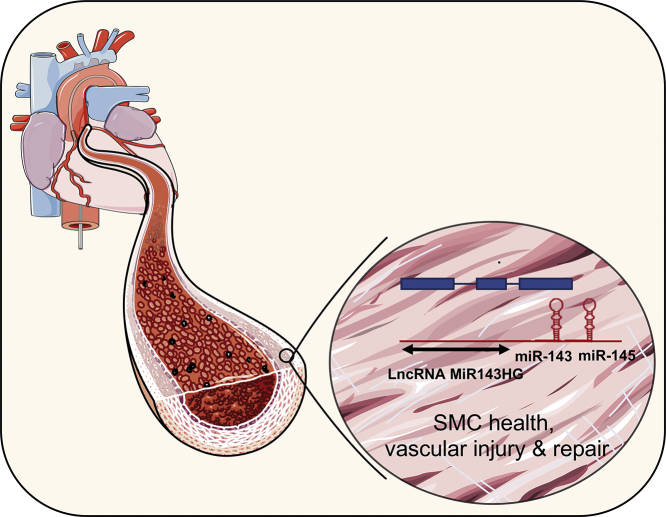

## 1 Introduction

In mammalian cells, >90% of transcripts are now known to code for a vast repertoire of noncoding RNAs, which range from ~22 nt (short noncoding RNAs) to long noncoding transcripts >200 nt in length [1,2]. Many studies have contributed to unravelling the complexity of the regulatory networks involving noncoding RNAs [3], and this is becoming an area of great interest to the field. However, their mechanistic role in regulating gene expression programs and their involvement in physiological and pathological conditions is still incompletely known and requires improved knowledge. This is particular relevant for cardiovascular development, homeostasis and disease context. To date, microRNAs represent the most studied class of small noncoding RNAs with well characterised biological function and a single mode of action [4]. Notably, many microRNA expression patterns have been associated with several human diseases, thus suggesting their influence in the cellular response to pathological stress [5]. How miRNAs are regulated, is however, a more complex question, as this can occur at the transcriptional and post-transcriptional level [6]. Recently, the crosstalk between miRNAs and long noncoding RNA (lncRNA) has attracted increasing attention leading to the identification of new regulatory networks (reviewed by Ballantyne et al. and Yoon et al. [7,8]). An example of such cross-talk is the interaction between lncRNA MiR143HG and miR-143/145; the focus of this review. Mir-143/145 is a vascular-enriched miRNA cluster, fairly extensively studied in vascular biology and in the pathophysiology of cardiovascular disease [[9], [10], [11], [12], [13]]. This microRNA cluster was shown to be encoded in a bicistronic unit [13] located downstream of the lncRNA MiR143HG, host gene of the miR-143 miRNA. Notably, the structure, the expression and the function of lncRNAs has gained increasing attention, resulting in rapid progression in the understanding of their potential importance [14,15].

The effort that has been dedicated toward the understanding of lncRNA biology and their characterisation, resulted in a more accurate genomic annotation, subcellular compartmentalisation, structural and mechanistic roles in physiological and pathological contexts thus revealing their functional heterogeneity. The eukaryotic ncRNA world is conventionally divided into two groups of transcripts based on the length of their sequence [2]: many of them are processed into small ncRNAs (<200 nt) including microRNAs, transcription initiation RNAs (tiRNAs), Piwi-interacting RNAs (piRNAs), small nuclear RNAs (snoRNAs), small interfering RNAs (endogenous siRNAs) [16]. The second group of ncRNA transcripts is represented by long ncRNAs, comprising transcripts longer then 200 nt in their mature form [17], of which only a small fraction have been characterised mechanistically to date.

## 2 MicroRNA biogenesis

MicroRNAs are negative regulators of gene expression at the post transcriptional level [18]. Their biogenesis is a two-step process which starts in the nucleus with the transcription of a pri-microRNA guided by RNA polymerase II or III, and ending in the cytoplasm (reviewed by Krol et al. and Davis-Dusenbery et al.) [19,20]. The primary transcript, originating in the nuclear compartment, is cleaved by RNAseIII (Drosha) to produce the precursor miRNA (pre-miRNA), and subsequently transported into the cytoplasm where it is processed by a specific endonuclease (Dicer) [21]. The result is a double-stranded RNA (guide and passenger strand), which is then integrated into the RNA-induced silencing complex (RISC). One strand is usually degraded, and the other strand will guide the RISC complex to the mRNA target. However, exceptions to this phenomenon are known. For example the passenger strand of the stem loop may be functional, as observed with let-7 family members [22] and miR-126 [23]. A specific sequence of the mature miRNA, called the seed sequence, is essential to recognise and bind the mRNA target, usually - but not exclusively- in its 3′ untranslated region (3’UTR). This phenomenon results in a negative regulation of gene expression by translational inhibition or destabilization and degradation of the mRNA target molecule [18].

## 3 LncRNA biology and function

**Fig. 1:**
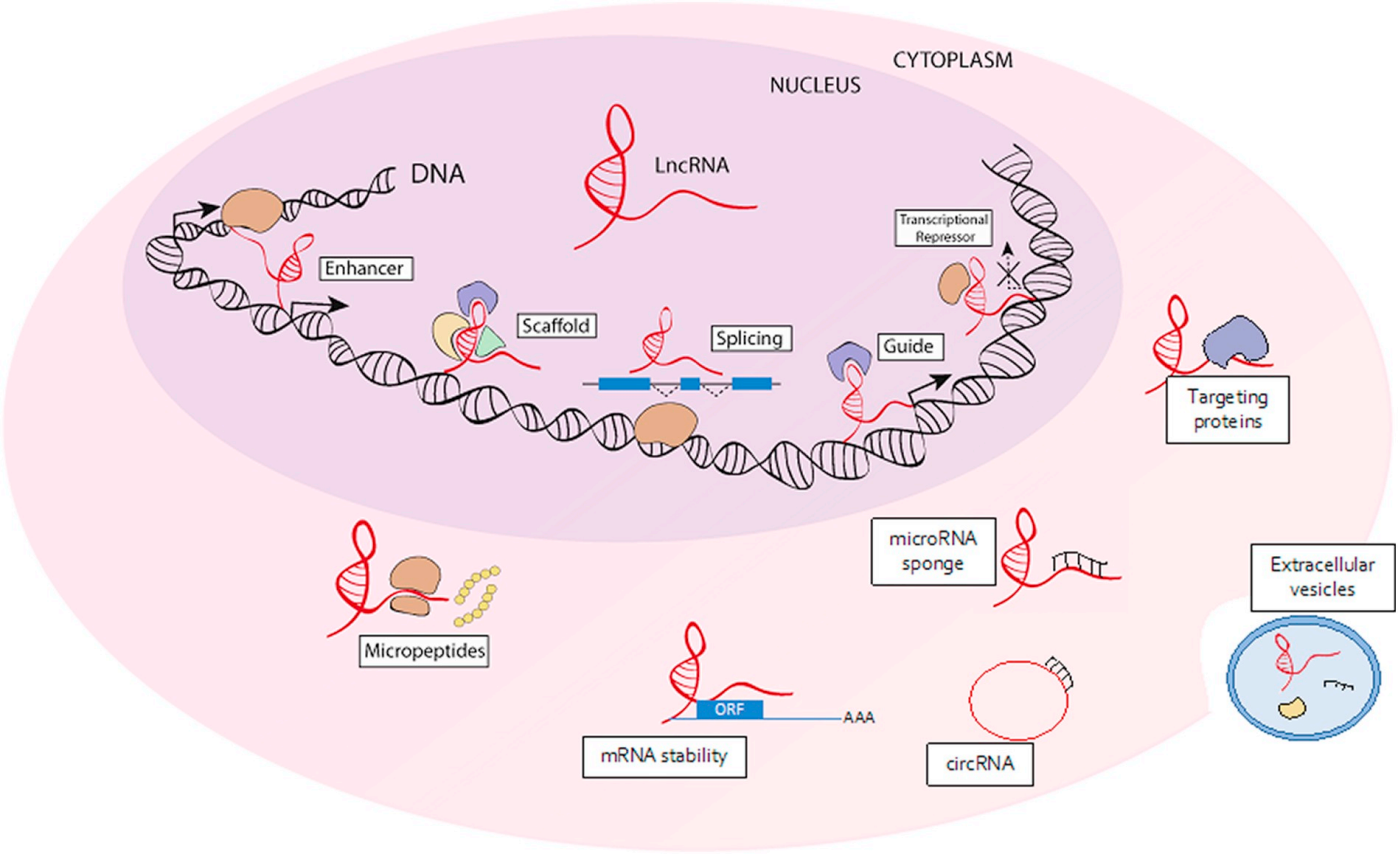
Long noncoding RNA function. In the nucleus, lncRNAs can modulate gene expression by enhancing or repressing the transcription of specific genomic loci by recruiting chromatin modifying complexes or inhibiting the binding of transcriptional factors to their DNA targets [39]. They can also modulate the splicing process by inducing or inhibiting the abundance of specific transcripts [14]. In the cytoplasm they can modulate gene expression at post-transcriptional level, interacting with proteins or other ncRNAs (microRNAs and mRNAs). Many lncRNA seem to act as scaffold for the assembling of proteins involved in same molecular networks [40]. Some of them can also regulate the processing of their mRNA including translation and degradation [41]. LncRNA can act as molecular sponges for microRNAs thus limiting their capability to bind their targets. LncRNA can also encode short functional peptides called (micropeptides) [42]. Recentely, lncRNA have also been described as important players of cell-cell communication being secreted in the extracellular environment by extracellular vesicles [43].

Long noncoding RNAs (lncRNAs) are a large family of ncRNAs commonly described as transcripts longer than 200 nt with limited or no open reading frame (ORFs) [24]. This relatively new class of transcripts was initially identified by large-scale sequencing of cDNA libraries in mouse [25]. Although the function of only 10% of the already annotated lncRNAs has been identified, the number of newly identified lncRNAs continues to rise with advances in techniques to characterise their structure and associated bioinformatical tools that are available [26]. Similar to protein-coding genes, lncRNAs are transcribed by RNA Polymerase II or III followed by modifications associated with capping, splicing, histone modifications and in some cases polyadenylation [27]. Notably, while they generally show low conservation levels of their primary sequence [28], the promoter of some lncRNAs was found to be more highly preserved across species [27]. Remarkably, even though the structure of relatively few lncRNAs have been described to date [29,30], it is becoming clear that lncRNAs often fold into thermodynamically stable structures. This knowledge may be instrumental in characterizing their function [31]. Different sub-groups of lncRNAs have been described based on their genomic location and orientation: sense, antisense, bidirectional, intronic, intergenic (lincRNA) and circular lncRNAs [32]. At a molecular level, the capability of interacting with multiple partners, such as RNA, DNA, or protein, endows them with several complex biological functions based on their subcellular localisation (reviewed by Ponting et al. and Ulitsky et al.) [15,33]. Particularly, their subcellular localisation now appears strictly connected to their functional role in modulating cellular processes (Fig. 1) [34]. Importantly, some of them have recently been described to have specific RNA-binding motives, critical for their nuclear localisation and the regulation of gene expression [35]. Nonetheless, one of the main features of lncRNA molecules is their cell- and temporal-specific expression profile, which can be modulated in response to physiological or pathological cues [36,37]. Moreover, the modulation of lncRNA expression in specific pathological contexts and the involvement of these molecules in cell-cell communication, has raised interest in investigating their potential role as biomarkers during vascular injury [38]. Deep sequencing and comparison between different pathological states has revealed profound changes in the expression of ncRNAs, indicating their role and involvement in the pathophysiology of disease. Therefore, deciphering their molecular mechanism of action in both, physiological and pathological contexts, might open new possibilities of therapeutic interventions.

Collectively, there is wealth of known and evolving data supporting the substantial influence that miRNA and lncRNA possess. In this review, we bring this together and focus on the biology and function of the lncRNA host gene of miR-143, miR-143HG and the miRNAs miR-143 and miR-145 and review their known roles in the cardiovascular system. We further highlight particular areas that we believe are important to assess in the future.

## 4 The biology of the miR-143/145-MiR143HG axis

### 4.1 miR-143/145 in vascular cell biology

**Fig. 2:**
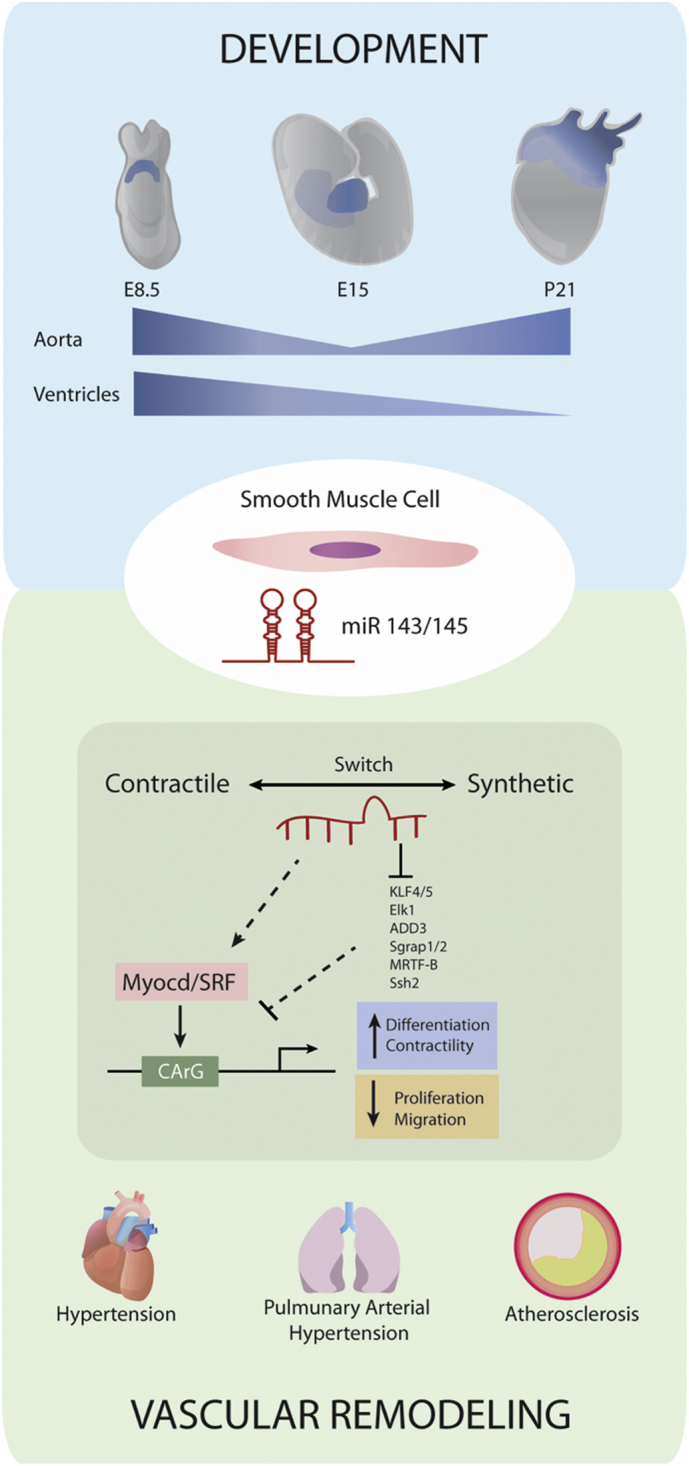
Regulatory networks controlled by miR-143/145 in cardiovascular development and disease in vSMCs. Schematic illustration of the main pathways regulated by miR-143/145 in vSMCs. During development, miR-143/145 show high expression levels in the myocardium of mouse embryo (E7-E9). In late cardiogenesis, expression mainly correlates with the ventricles and atria (E15). The expression pattern is then inverted post-natally, where miR-143/145 is expressed only in the aorta and pulmonary arteries. In physiological conditions in adult mouse, the vascular-specific microRNA cluster controls the transcription of contractility and differentiation genes typical of a SMC signature (α-SMA, Calponin, Mhy11). Particularly, the transcriptional regulation of vSMC contractile phenotype involves binding of SRF/Myocd (Serum Responsive Factor/Myocardin) to the CArG box located on the promoter of contractility genes, which in turn induce the miR-143/145 expression. Importantly, miR-143/145 is also implicated in the regulation of the vSMCs phenotypic switch in vascular disease. Particularly, miR-143/145 was found dysregulated in response to vascular injury, such as hypoxia, stretch, shear stress, growth factors, pro-inflammatory cytokines, inducing genes regulating vSMC proliferation, migration and plasticity [12].

MiR-143 and miR-145 are among the best characterised small regulatory ncRNAs in the vasculature. The underpinning biogenesis and processing of the miR-143/145 cluster has been extensively studied by different research groups [4,20,44]. Collectively, these findings show that miR-143/145 are co-transcribed from a single bicistronic unit, localised on human chromosome (chr) 5 and on murine chr18 (GRCh38) to an individual pri-microRNA transcript. Therefore miR-143/145 share regulatory elements of their expression [9,10]. Studies on sequence conservation have revealed the presence of a highly conserved region (4.2 kb) of the promoter of the miR-143/145 cluster, containing binding sites for transcriptional factors regulating vascular smooth muscle cell (vSMC) contractility. Particularly, the serum response factor (SRF)/myocardin (Myocd) complex is a potent activator of vSMC differentiation (Fig. 2) [45]. Upon binding of the CArG-box on the miR-143/145 promoter, the SRF/Myocd axis induces tissue-specific microRNA expression in the heart and the vasculature [10,13] [11]. In addition to the SRF/Myocd pathways, the regulation of the miR-143/145 expression is controlled by transforming growth factor β 1 (TGF-β1)/bone morphogen protein 4 (BMP4) network [46,47]. This family of growth factors induces the transcription of genes associated to the vSMC contractile phenotype including the miR-143/145 expression, which in turn represses the expression of genes associated with vSMC phenotypic switch [11]. However, Boettger et al. identified angiotensin-converter enzyme (ACE) as a critical regulator of vSMC contractility and as a putative target modulated by miR143/145-mediated translation repression [48]. Likewise, Jag-1/Notch, a known regulatory pathway of vSMC phenotype [49] was described as a positive regulator of miR143/145 expression. This pathways seems to act independently from SRF/Myocardin, thereby amplifying the vSMC differentiation signals [50]. Boettger et al. and Elia et al. studied the miR-143/145 expression profile at different stages of mouse embryonic development [48,51]. In both studies, miR-143/145 was found to be highly expressed in the heart at early stage of embryogenesis (E9-E16) while, postnatally, the expression of miR-143/145 strongly correlates with vSMC in the aorta and coronary vessels [48,51,52]. This dynamic expression might allow the proliferation and maturation necessary during embryogenesis, once a vSMC signature is established in early development (Fig. 2). Although miR-143 and miR-145 are co-transcribed in a bicistronic primary transcript, studies have demonstrated the absence of homology in their mature sequence, indicating their capability to bind and regulate different targets [12]. For instance, miR-145, but not miR-143, was described to play a key role in controlling embryonic stem cells (ESC) differentiation, by directly repressing critical pluripotency factors: OCT4, SOX2, KLF4 [53]. Moreover, studies on heart morphogenesis in zebrafish, demonstrated the importance of miR-143 as intrinsic factor involved in the cardiac development, whereas mir-145 did not show a similar function. Particularly, in vivo knockdown (KD) of miR-143 in zebrafish, showed that miR-143 acts as a critical regulator of myocardial cell growth and elongation by directly targeting Adducin3 (Add3) protein which blocks actin filaments rearrangement [54]. Moreover, miR-143 KD caused myocardial abnormalities in atrial and ventricular structure and defects in heart function [55]. Bioinformatic approaches and in vitro experiments performed in human and mouse vSMCs identified two transcriptional factors, ETS domain-containing protein (Elk1) and Myocd, as direct targets for miR-143 and miR-145 respectively. Particularly, Elk1, myogenic repressor and inductor of vSMC proliferation [56], was found to be transcriptionally downregulated by miR-143 and upregulated in the presence of an antagomir for miR-143 in vSMCs [13]. On the other hand, miR-145 is important for the maintenance of vSMC phenotype by increasing the levels of Myocd which in turn induces vSMC differentiation and contractility [13]. Furthermore, binding sites for miR-145 were found in the 3’ UTR of Kruppel-like factor 4 (Klf4), positive regulator of vSMC proliferation and phenotypic switch.

Interestingly, despite the lack of clear sequence homology, the two microRNAs were also described to target common genes and pathways such as Slit-Robo GTPase-activating protein 1 and 2 (Srgap1 and 2) and Sling-shot 2 (Ssh2) involved in the regulation of F-actin polymerization and depolymerization, cell migration and cytoskeleton remodelling [12]. Importantly, these studies reveal complex regulatory networks in which miR-143 and miR145 can act synergistically in the reinforcement of vSMC contractile phenotype by independently targeting different molecular pathways.

### 4.2 miR143/145 in cardiovascular disease (CVD) pathogenesis

Since miR-143/145 are critical in the maintenance of vSMC contractility and differentiation, here we describe their role in vSMC-driven pathologies, such as hypertension, atherosclerosis, and pulmonary arterial hypertension (PAH). Several studies have interrogated their involvement in modulating the vSMC response to vascular injury [9,10,13,49,51] [58,59]. Neointima formation is one of the main processes underlying vascular remodelling following vascular damage [60]. In vivo studies using miR-143 and miR-145 double KO mice reported that in the KO animals had formation of neointimal lesions in the femoral artery compared to wild type animals, even in the absence of injury. This suggests the critical role of these microRNAs in controlling the vSMC phenotypic switch during vascular injury [48]. In line with these results, miR-143 and miR-145 were found to be dramatically downregulated in the carotid artery of balloon-injured rats, where proliferative and dysregulated vSMC contribute to the lesion development [58]. In addition, adenovirus-mediated overexpression of the miR-145 stem-loop inhibited the formation of neointima in balloon-injured arteries [52]. Moreover, lentivirus-mediated overexpression of miR-145 in Apoliprotein E (ApoE^−/−^) KO mice was found to reduce atherosclerotic plaque size and induce a more stable plaque phenotype, as indicated by a significant increase in the fibrous cap area and plaque collagen content, and to decrease in the number of pro-inflammatory macrophages populating the plaque [61]. In contrast, in human, the miR-143/145 cluster was significantly upregulated in advanced and symptomatic atherosclerotic plaques compared to asymptomatic controls, probably indicating the dynamic expression of the microRNA cluster during the late stages of the pathology progression [62].

In contrast to the reduction in expression of miR-143 and miR-145 commonly observed in the peripheral vascular system in response to injury, miR-143 and miR-145 were found to be upregulated in a hypoxia-induced mouse model of PAH and in human clinical samples versus healthy controls. This suggest these miRNAs affect the pathophysiology of PAH via increased expression. Consistent with this notion, anti-miR-mediated knockdown of miR-145 in mouse resulted in a protective effect from the development of PAH. Consistent with these results, miR-145 was found upregulated in arterial SMCs of patients affected by idiopathic and familiar PAH versus controls, thus confirming the involvement of the microRNA in the pathophysiology of PAH vascular remodelling [9]. Importantly, further in vivo and in vitro studies interrogating the role of miR-143 during the development of PAH, showed that miR-143 was selectively upregulated during pulmonary arterial smooth muscle cell (PASMC) migration. Moreover, the modulation of miR-143 altered the migratory capability and apoptotic rate of PASMCs and, remarkably, the genetic ablation of miR-143 alleviated the development of PAH in vivo [10].

Collectively, these studies demonstrate that both miR-143 and miR-145 have important functions in diverse vascular beds, that their expression can be regulated in either direction in response to diverse injury stressors and that intervention to modulate levels of these miRNAs is often therapeutic.

### 4.3 miR143/145 as biomarkers of CVD

MiRNAs have also been proposed as potential biomarker for cardiovascular diseases since their dysregulation in pathological stress conditions is quite clear, and miRNA are often released from cells and tissues in response to injury. With this in mind, circulating miR-145 was found upregulated in the plasma of patients with unstable versus stable angina [57,59]. Importantly, it was reported by several studies that extracellular vesicles are carriers of ncRNAs in cardiovascular disease [63,64]. In this context, miR-143 was identified as a potential paracrine mediator of cell-cell communication between SMC endothelial cells (ECs) during vascular remodelling in PAH [10]. Importantly, the SMC-EC signalling carried out by miR-143/145 in extracellular vesicles, was described as atheroprotective in ApoE^−/−^ mice as it contributed to the reduction of plaque development [65]. The transfer of miR-143/145 was described to be mediated not only by extracellular vesicles, but also by membrane protrusions between SMCs and ECs. These findings were confirmed by in vitro and in vivo approaches which showed that, once being transferred from SMCs, miR-143 and miR-145 were able to modulate EC angiogenesis and proliferation by directly binding hexokinase II and integrin β 8 respectively [46]. Collectively, these results advanced the knowledge of the complex regulatory circuits involving miR-143 and miR-145, defining their involvement as modulators of vSMC phenotype in physiological conditions and vascular disease. Therefore, the molecular mechanisms regulating their expression may be a suitable target for therapeutic approaches. It will be critical, however, to ensure selective modulation of these miRNAs in the target tissue, since widespread modulation of these miRNAs might evoke unacceptable side effects.

### 4.4 The lncRNA MiR143HG: structure and functional importance

**Fig. 3:**
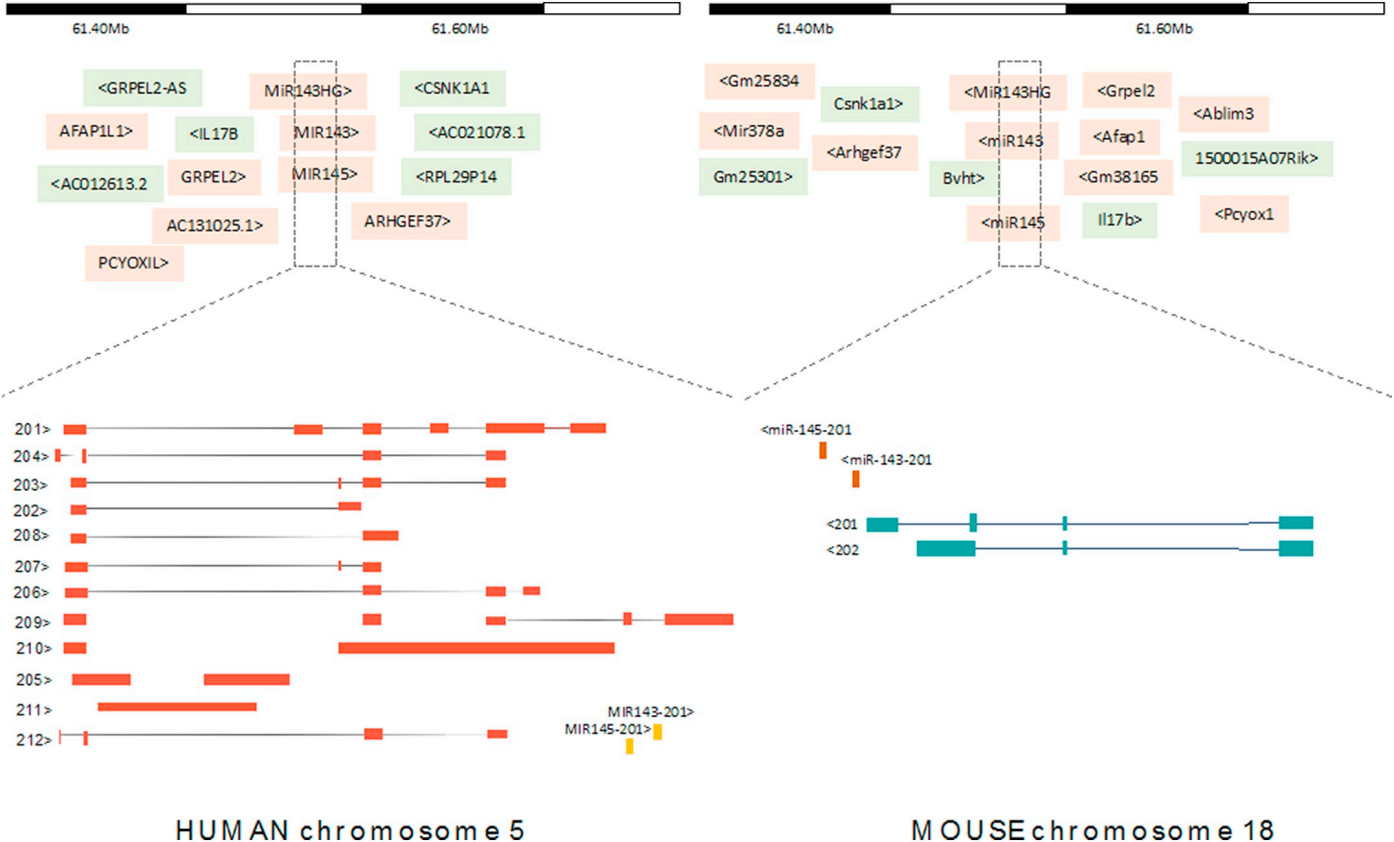
Schematic representation of human MiR143HG (Chr5 q32) and mouse (Chr18 E1) splice variants and pre-miRNA 143/145. The MiR143HG locus encodes for twelve lncRNA isoforms in human and two in mouse.

The lncRNA MiR143HG was first described in the literature by Ouzain et al. as differential expressed during cardiovascular lineage specification in cardiac precursor cells (CPCs) and proliferating cells [66]. Computational analysis classified MiR143HG among the transcripts lacking of any coding potential [67]. Moreover, ChiP-Seq analysis of human foetal and adult heart associated the transcript with an active cardiac enhancer [68]. Therefore, the lncRNA MiR143HG was named Cardiac Mesoderm Enhancer-associated Noncoding RNA (CARMN). For simplicity, here we will refer to the lncRNA as MiR143HG. MiR143HG belongs to the intergenic lncRNA group of ncRNAs with multi-exonic spliced variants. Publicly available datasets report the expression of MiR143HG mostly in the nucleus of different cell lines and organs [69] indicating the potential wide involvement of this lncRNA in different physiological contexts. Moreover, the genomic annotation in Ensembl includes 12 noncoding human transcripts generated by the MiR143HG locus, which have been recently confirmed by the RNA Capture Long-read Sequencing technique [70](Fig. 3). However, the potentially incomplete nature of data in the lncRNA annotation may ultimately lead to further updates to the annotation, which might especially true for variants that are rare. Importantly, the promoter region of MiR143HG shows evolutionary conservation across species (gene annotation set from GENCODE version 27), suggesting a potential conservation of the transcriptional regulation. Similarly to the human locus, murine MiR143HG is the host gene for the miR-143/145 cluster and yields two MiR143HG splice variants (mouse genome assembly GRCm38) (Fig. 3). Interestingly, analysis of chromatin for enhancer-associated noncoding RNA showed that MiR143HG may be implicated in the regulation of chromatin state activation and enhancer-like signature [71] suggesting a cis-acting function in cardiac differentiation.

### 4.5 LncRNA MiR143HG in the pathophysiology of disease

Importantly, MiR143HG is expressed not only during cardiac differentiation, but also in the adult mouse and human heart [72]. Particularly, Ouzain et al. described high expression of three of the twelve human isoforms in CPCs versus proliferating cells using bioinformatics and qPCR. Interestingly, the two downstream microRNAs, miR-143 and miR-145, were found to have opposite expression patterns from the lncRNA during cardiogenesis, thus indicating possible feedback mechanisms relating to transcript expression. Bioinformatic analysis conducted by super enhancer track and ChIP-analysis revealed that the lncRNA transcripts mapped to a cardiac enhancer, suggesting a role in cardiac specification and differentiation. Consistent with this observation, analysis on chromatin state revealed an active super-enhancer signature during cardiogenic differentiation between mesoderm and CPC, in which the expression of MiR143HG isoforms was found to be upregulated [50]. Furthermore, MiR143HG was also found to be highly expressed in mouse CPC, indicating a potential role during the differentiation of mouse ES. In vitro experiments in mouse and human foetal CPCs, showed that the depletion of MiR143HG expression by shRNAi or a GapmeR was associated with a significant reduction of cardiac differentiation markers, impairing CPC differentiation [66]. Aside from a role in physiology, the same research group demonstrated that MiR143HG expression was also influenced by cardiac stress in a murine model of myocardial infarction, where the two mouse transcripts were found upregulated. Moreover, the lncRNA was shown to be upregulated during cardiac remodelling in patients affected by aortic stenosis [66], thus suggesting its potential role during the response to the pathological remodelling in human and mouse heart. The lncRNA was also was described to be involved in the differentiation of CPC into cardiomyocytes or smooth muscle lineage being targeted by NOTCH signalling [73]. Plaisance et al. observed an increase of MiR143HG expression in CPC producing smooth muscle cells, were the microRNA cluster was found to be expressed at high levels suggesting a positive co-regulation of the two ncRNAs. In agreement with these results, the gapmeR-mediated knock-out of MiR143HG induced a significant downregulation of miR-143 and miR-145 and a specification of CPCs toward the cardiomyocyte commitment. Importantly, it was demonstrated that MiR143HG and miR-143/145 are involved in a complex molecular circuit regulating CPC differentiation into SMC. NOTCH activates MiR143HG, a proximal enhancer element upstream of miR-143 and miR-145, which in turns modulate the miR143/145 cluster, thus inducing smooth muscle differentiation genes [73].

## 5 Conclusions

NcRNAs can regulate gene expression at different levels, i.e. transcriptionally, post-transcriptionally and post-translationally; and regulate various types of cell function(s): differentiation, metabolism, and stress-responses [3]. MiR-143 and miR-145 have been well characterised as crucial modulators of vSMC physiology, being central in the regulation of complex molecular networks modulating vSMC proliferation, migration, apoptosis and a phenotypic switch [46,51,61,74]. Importantly, dysregulated expression was associated with the development of cardiovascular diseases [10,11,13]. More recently, increasing evidence supports a critical function for lncRNA in the regulation of pathophysiological processes in the cardiovascular system development and the progression of cardiovascular disease [36,75,76]. MiR143HG, host gene for miR-143 and miR145 and uncommonly well conserved across species, was described to be important during cardiac development and to be dysregulated under pathological conditions in cardiomyocytes [66,73]. Recent studies have investigated the extensive network of interactions between ncRNAs, microRNAs and lncRNA, forming crucial regulatory axis involved in the modulation of cell function [7,8]. MiR143HG and miR-143/145 represent a significant example of a lncRNA/microRNA axis involved in the regulation of vSMC function [77]. However, the mechanism of action by which this lncRNA axis may regulate cellular processes still remains unclear. Certainly, further investigations are required to identify the causal mechanism that governs the phenotypic effects observed in vitro and in vivo. The genetic ablation of MiR143HG in an animal model would represent a comprehensive tool to elucidate the functional consequence of MiR143HG loss in the development in early life and progression of cardiovascular diseases in the adult. It is necessary to mechanistically dissect the lncRNA/microRNA axis by separately modulating the expression of the three ncRNAs through targeted disruption of the axis. Given the high level of complexity of the MiR143HG locus, which suggests the essential role and interplay of MiR143HG-miR143/145 axis, additional investigation is required to clearly define their molecular interactions and their functional role in cardiovascular disease development and progression.
